# Tristetraprolin Regulates T_H_17 Cell Function and Ameliorates DSS-Induced Colitis in Mice

**DOI:** 10.3389/fimmu.2020.01952

**Published:** 2020-08-14

**Authors:** Hui Peng, Huan Ning, Qinghong Wang, Jinping Lai, Lin Wei, Deborah J. Stumpo, Perry J. Blackshear, Mingui Fu, Rong Hou, Daniel F. Hoft, Jianguo Liu

**Affiliations:** ^1^Division of Infectious Diseases, Allergy and Immunology, Department of Internal Medicine, Saint Louis University School of Medicine, Saint Louis University, St. Louis, MO, United States; ^2^Department of Pathology, Saint Louis University School of Medicine, Saint Louis University, St. Louis, MO, United States; ^3^Department of Immunology, School of Basic Medicine, Hebei Medical University, Shijiazhuang, China; ^4^National Institute of Environmental Health Sciences, Research Triangle, NC, United States; ^5^Shock/Trauma Research Center and Department of Basic Medical Science, School of Medicine, University of Missouri-Kansas City, Kansas City, MO, United States

**Keywords:** tristetraprolin, RNA-binding protein, IL-17, mRNA decay, T_H_17, DSS, colitis

## Abstract

T_H_17 cells have been extensively investigated in inflammation, autoimmune diseases, and cancer. The precise molecular mechanisms for T_H_17 cell regulation, however, remain elusive, especially regulation at the post-transcriptional level. Tristetraprolin (TTP) is an RNA-binding protein important for degradation of the mRNAs encoding several proinflammatory cytokines. With newly generated T cell-specific TTP conditional knockout mice (CD4^Cre^TTP^f/f^), we found that aging CD4^Cre^TTP^f/f^ mice displayed an increase of IL-17A in serum and spontaneously developed chronic skin inflammation along with increased effector T_H_17 cells in the affected skin. TTP inhibited T_H_17 cell development and function by promoting IL-17A mRNA degradation. In a DSS-induced colitis model, CD4^Cre^TTP^f/f^ mice displayed severe colitis and had more T_H_17 cells and serum IL-17A compared with wild-type mice. Furthermore, neutralization of IL-17A reduced the severity of colitis. Our results reveal a new mechanism for regulating T_H_17 function and T_H_17-mediated inflammation post-transcriptionally by TTP, suggests that TTP might be a novel therapeutic target for the treatment of T_H_17-mediated diseases.

## Introduction

T_H_17 cells play a pivotal role in the pathogeneses of several diseases, including autoimmune arthritis, multiple sclerosis, and inflammatory bowel disease (IBD) (1, 2). Differentiation of T_H_17 cells requires T-cell receptor (TCR) signals plus transforming growth factor β (TGF-β) and interleukin 6 (IL-6) stimulation. Several transcription factors, including RORγt, Stat3 and interferon-induced factor 4 (IRF-4), have been shown to mediate T_H_17 cell differentiation (3–7). In addition, IL-23 is essential for T_H_17 cell survival/expansion and for generation of pathogenic T_H_17 cells, although initial differentiation of T_H_17 cells depends on IL-6 and TGF-β stimulation (8). As the signature cytokine produced by T_H_17 cells, IL-17 contributes to the pathogenesis of T_H_17-mediated inflammatory diseases, such as psoriasis (9), rheumatoid arthritis (10), and IBD (11, 12), as well as host defense against certain pathogens (13). Therefore, tightly controlling the development and function of T_H_17 cells is essential for maintaining homeostasis. So far, most studies have focused on transcriptional regulation of T_H_17 cell differentiation and function; much less is known about how T_H_17 cells are regulated at the post-transcriptional level.

Tristetraprolin (TTP, also known as TIS11, ZFP36, and Nup475), a CCCH tandem zinc-finger protein (ZFP) coded by gene Zfp36, is involved in the regulation of inflammatory responses at the post-transcriptional level (14). Expression of TTP mRNA and protein is tightly regulated in macrophages under the control of TLR4 and other TLR signaling (15–17). Upregulation of TTP can reduce inflammatory responses in macrophages (18). TTP binds to AREs within the 3′ untranslated region (3′ UTR) of its target transcripts, causing destabilization of the mRNAs encoding tumor necrosis factor α (TNF-α) (19), granulocyte-macrophage colony-stimulating factor (GM-CSF) (20), cyclooxygenase 2 (21), IL-2 (22), IL-10 (23), and the chemokine CXCL1 (24), among others (25). The mRNAs encoding TNF-α and GM-CSF are stabilized in TTP-deficient mice and in cells derived from them (16, 20). Oversecretion of these cytokines in TTP knockout (KO) mice results in a severe systemic inflammatory response including arthritis, autoimmunity, and myeloid hyperplasia (26). We previously demonstrated that TTP inhibits IL-23 expression through promoting p19 mRNA degradation via AREs in the 3′ UTR (27). Molle et al. (28) found similar findings as ours and showed that IL-23 oversecretion in conventional TTP^–/–^ mice causes an increase in T_H_17 cells, and both IL-23 and IL-17A contribute to the chronic inflammation in conventional TTP^–/–^ mice. Although TTP is one of the best characterized post-transcriptional regulators and ARE binding proteins, it remains largely unclear whether TTP affects T cells, specifically T_H_17 cell development and function *in vivo*.

In this study, we generated T cell-specific TTP conditional KO (CD4^Cre^TTP^f/f^) mice to investigate the effects of TTP on T-cell development and function. Aging CD4^Cre^TTP^f/f^ mice developed spontaneous skin inflammation and displayed an increase in systemic IL-17A and skin T_H_17 cells. CD4^+^ T cells lacking TTP were more likely to develop into T_H_17 cells compared with wild-type (WT) CD4^+^ T cells. In fact, IL-17 productivity was enhanced in TTP^–/–^ CD4 T cells compared with WT CD4^+^ T cells at the single-cell level. This increased IL-17 production in TTP^–/–^ CD4^+^ T cells was mediated by increased IL-17A mRNA stability, demonstrating that TTP promotes the degradation of IL-17A mRNA. Furthermore, the CD4^Cre^TTP^f/f^ mice were prone to DSS-induced colitis with higher levels of serum IL-17A and T_H_17 cells in mesenteric lymph node (LN) than WT mice. Neutralization of IL-17A reduced the severity of colitis in CD4^Cre^TTP^f/f^ mice. Therefore, our study reveals a novel post-transcriptional pathway through which TTP suppresses the function of T_H_17 cells.

## Results

### T Cell-Specific TTP Conditional KO Mice Develop Chronic Skin Inflammation During the Aging Processes

**FIGURE 1:**
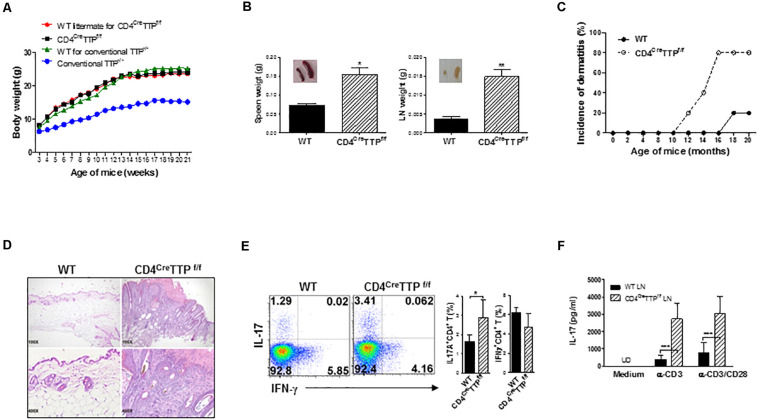
Aging mice with specific deletion of TTP in T cells develop chronic skin inflammation. **(A)** Growth curves of CD4^Cre^TTP^f/f^ mice and wild-type (WT) littermates were monitored and recorded weekly. Data are presented as mean ± SD of 6 mice in each group. **(B)** Data represent weight (g) of spleens and LN (means ± SD) from three CD4^Cre^TTP^f/f^ mice and WT littermate at 11–13 months old. Inserted images were spleen and lymph node of CD4^Cre^TTP^f/f^ mice and WT littermates. **(C)** Percentages of dermatitis. Five mice in each group. **(D)** Skin sections from CD4^Cre^TTP^f/f^ mice and WT mice were analyzed with hematoxylin and eosin staining. Cells from draining lymph nodes (LN) of the affected skin in five WT and CD4^Cre^TTP^f/f^ mice were stimulated with PMA and ionomycin for 4 h and analyzed by flow cytometry for intracellular cytokine gated on CD4^+^ T cells **(E)**, or stimulated with anti-CD3 (1 μg/mL) with or without anti-CD28 (1 μg/mL) Abs for 3 days for detection of IL-17A by ELISA **(F)**. Data shown are means ± SD from five mice. UD, undetectable. *p < 0.05, **p < 0.01, and ***p < 0.001 between groups.

To determine the effects of TTP on T cells, we generated T cell-specific TTP conditional KO mice by crossing TTP^flox/flox^ mice with mice expressing Cre recombinase transgene driven by the CD4 promoter (CD4^C*re*^) (29). Cre recombinase led to deletion of the exon 2 and the 3′ UTR of TTP in both CD4 and CD8 T cells due to CD4/CD8 coexpression transiently in the thymus (30). Exon 2 codes the tandem zinc-finger domain, which is responsible for the RNA-binding activity of TTP. Functional TTP KO specifically in CD4 T cells was evidenced in CD4 T cells lacking TTP expression and in macrophages with normal TTP expression (Supplementary Figure 1A). The percentages of thymic CD4 and CD8 T cells were similar between WT and CD4^Cre^TTP^f/f^ mice (Supplementary Figure 1B), and the numbers of macrophages and dendritic cells (DCs) in spleen were comparable between WT and CD4^Cre^TTP^f/f^ mice (Supplementary Figure 1C). Among most obvious phenotypes of the conventional TTP^–/–^ mice are growth retardation and joint swelling (26). Somewhat surprisingly, the conditional CD4^Cre^TTP^f/f^ mice grew normally, with body weights that were identical to those of their WT littermates and with no signs of joint inflammation (Figure 1A). Enlargement of LNs and spleens was noted in the CD4^Cre^TTP^f/f^ mice (Figure 1B). Although the younger CD4^Cre^TTP^f/f^ mice did not show any signs of chronic inflammation, CD4^Cre^TTP^f/f^ mice started showing evident dermatitis within 10 months of birth, and 80% had dermatitis by 16 months of age (Figure 1C). Histological analysis of the affected skin indicated that the epidermal layer thickness was significantly increased, and more inflammatory cells were present in the affected skin (Figure 1D). Immunofluorescence staining showed an increase in IL-17^+^CD4^+^ cells in the skin lesions of CD4^Cre^TTP^f/f^ mice compared to WT mice (Supplementary Figure 1D). Next, we isolated cells from draining LNs of the affected skin and stimulated them with PMA and ionomycin for 4 h, followed by detection of IL-17– and interferon γ (IFN-γ)–producing CD4 T cells by flow cytometry. As shown in Figure 1E, there were higher percentages of IL-17–producing CD4 T cells in the draining LNs of CD4^Cre^TTP^f/f^ mice than in WT mice. When these LN cells were stimulated by anti-CD3 with or without anti-CD28 antibody (Ab), more IL-17A was secreted by LN cells of CD4^Cre^TTP^f/f^ mice than that of WT mice (Figure 1F), suggesting a correlation between IL-17 and the development of dermatitis in the aging CD4^Cre^TTP^f/f^ mice.

### T Cell-Specific TTP Conditional KO Mice Have More IL-17–Producing Effector T Cells

**FIGURE 2:**
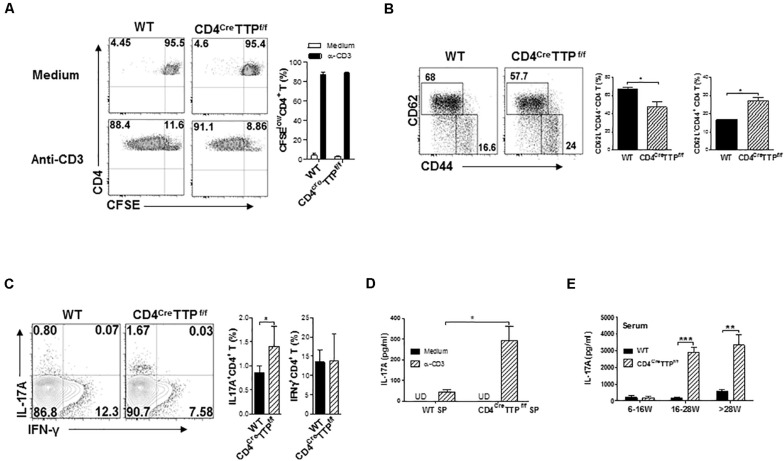
T cell-specific TTP conditional knockout mice have increased IL-17–producing effector T cells. **(A)** Single spleen cells of wild-type (WT) and CD4^Cre^TTP^f/f^ mice aged 6–8 months were labeled with CFSE and cultured with anti-CD3 (1 μg/mL) Ab for 4 days before the proliferation was assessed by flow cytometry. Percentages of CFSE^*low*^ CD4^+^ T cells were summarized from three to four independent experiments. **(B)** Wild-type and CD4^Cre^TTP^f/f^ splenocytes were stained for CD44 and CD62L gated on CD4^+^ cells. Percentages of CD62L^–^ CD44^+^ (effector) and CD62L^+^CD44^–^ (naive) CD4^+^ T cells from four independent experiments were summarized and compared by *t*-test. **(C)** WT and CD4^Cre^TTP^f/f^ splenocytes were stimulated by PMA and ionomycin for 4 h and then analyzed by flow cytometry for intracellular cytokine gated on CD4^+^ T cells. Fluorescence-activated cell sorting images represent one of five similar results. Percentages of IL-17^+^ and IFN^+^ CD4 T cells from four independent experiments were summarized and compared by *t*-test. **(D)** Cells from spleens (SP) of WT and CD4^Cre^TTP^f/f^ mice were stimulated with anti-CD3 Ab (1 μg/mL) for 3 days. Supernatants were collected and filtered for detection of IL-17A by ELISA. Data represent means ± SD from three to four mice in each group. **(E)** IL-17A in serum of WT and CD4^Cre^TTP^f/f^ mice aged between 6 and 30 weeks old was determined by ELISA. Data in E and F are expressed as means ± SD, with four to five mice per group. *p < 0.05, **p < 0.01, and ***p < 0.001 between groups.

T cells, especially T_H_17 cells, are major producers of IL-17. To test whether TTP affected T_H_17 cell function, we first checked CD4 T-cell proliferation. The proliferative capacity of CD4 T cells was similar between CD4^Cre^TTP^f/f^ mice and WT mice (Figure 2A). However, CD4^+^ T cells from CD4^Cre^TTP^f/f^ mice (Figure 2B) and from conventional TTP^–/–^ mice (Supplementary Figure 2A) were more likely to become CD62L^–^ CD44^+^ effector T cells compared with cells from WT mice, indicating that T cell-specific TTP deficiency leads to CD4 T-cell activation. Indeed, CD4^+^ T cells from spleens of CD4^Cre^TTP^f/f^ mice secreted higher levels of IL-17A than WT cells (Figures 2C,D). Systemic IL-17A levels were also significantly elevated in CD4^Cre^TTP^f/f^ mice compared with their WT littermates (Figure 2E). Interestingly, the increased serum IL-17A was not manifest until CD4^Cre^TTP^f/f^ mice were older than 16 weeks (Figure 2E). CD4 T cells purified from spleens of the conventional TTP^–/–^ mice also showed a significant increase in IL-17–producing effector CD4 T cells when the mice were older than 8 months of age (Supplementary Figure 2B). In addition, the levels of IL-17 and IL-6 in culture supernatants of CD4^+^ T cells (Supplementary Figure 2C) and IL-17A in serum (Supplementary Figure 2D) were increased significantly in conventional TTP^–/–^ mice compared with WT mice. These data indicate that TTP plays a role in suppression of IL-17 secretion and in T_H_17-mediated inflammation in aging mice.

### T_H_17 Cells Lacking TTP Have Increased per Cell Cytokine Productivity

**FIGURE 3:**
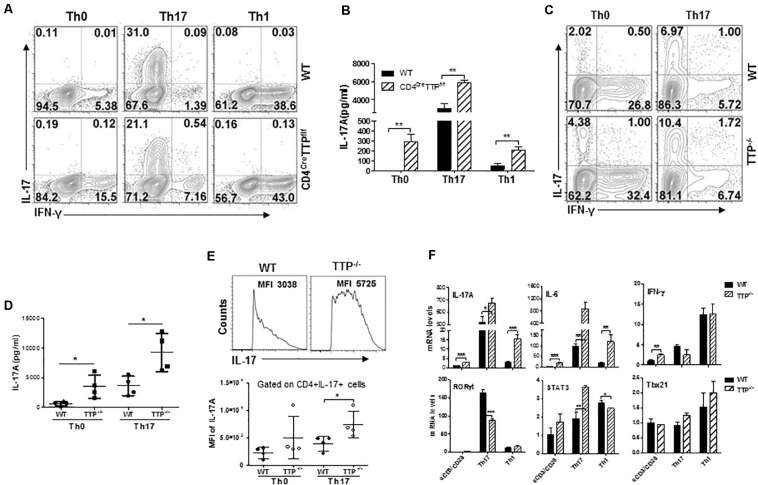
CD4 T cells deficient in TTP produce more IL-17 than wild-type (WT) T cells at the single-cell level. **(A)** Naive CD4^+^ T cells from CD4^Cre^TTP^f/f^ and WT mice aged 6–8 months were cultured under T_H_1 or T_H_17 polarizing conditions in 96-well plate coated with anti-CD3 (2 μg/mL) and anti-CD28 (2 μg/mL) Abs for 3 days before stimulation with PMA and ionomycin for 4 h. Intracellular IL-17A and IFN-γ in CD4^+^ T cells were analyzed by fluorescence-activated cell sorting (FACS). **(B)** Supernatants of the above cells were collected to measure IL-17A protein levels by ELISA (means ± SD from three independent experiments). **(C,D)** Total CD4^+^T cells from WT and TTP^– /–^ mice were stimulated with anti-CD3/CD28 Abs under T_H_17 polarizing conditions for 3 days, rested for 3 days, and then treated with P/I for 4 h, followed by FACS analysis **(C)**. IL-17A levels in the supernatants of the above cells were detected by ELISA and summarized from four independent experiments **(D)**. **(E)** Mean fluorescence intensity (MFI) of intracellular IL-17A in CD4^+^ T cells from WT and TTP^– /–^ mice under T_H_17 cell differentiation. Each dot represents one experiment. **(F)** IL-17A, IL-6, IFN-γ, *Rorc*, *Stat3*, and *Tbx21* mRNA expression in CD4 T cells under T_H_1 and T_H_17 cell differentiation for 4 days. Data shown are means ± SD from three independent experiments. *p < 0.05, **p < 0.01, and ***p < 0.001 between groups.

To figure out whether TTP deficiency could enhance T_H_17 cell differentiation, we differentiated naive CD4 T cells from WT and CD4^Cre^TTP^f/f^ mice into T_H_1 and T_H_17 subsets under T_H_1 and T_H_17 polarizing conditions and then measured intracellular IFN-γ and IL-17A with flow cytometry. IFN-γ–producing CD4 T cells were comparable between TTP^–/–^ CD4 T cells and WT CD4 T cells under T_H_0, T_H_1, and T_H_17 polarizing conditions (Figure 3A and Supplementary Figure 3A). Surprisingly, even the percentages of differentiated T_H_17 cells were comparable between TTP^–/–^ CD4 T cells and WT CD4 T cells (Figure 3A and Supplementary Figure 3A); the secretion of IL-17 by TTP^–/–^ CD4 T cells was increased under all conditions (Figure 3B). In addition, when total CD4 T cells from WT and TTP^–/–^ mice were cultured under T_H_0 and T_H_17 conditions, there was little increase of IL-17–producing CD4 T cells in cells lacking TTP (Figure 3C and Supplementary Figure 3B). This little increased TTP^–/–^ T_H_17 cells was in contrast to significantly increased levels of IL-17A produced by the TTP^–/–^ CD4 T cells in culture supernatants (Figure 3D). These data suggest that the increased IL-17 secretion by TTP^–/–^ CD4^+^ T cells may not be due to an increase in T_H_17 cell differentiation. Indeed, the mean fluorescence intensity of IL-17A was significantly increased in TTP^–/–^ CD4^+^ T cells compared with WT cells under T_H_17 differentiation conditions (Figure 3E), indicating that each TTP^–/–^ CD4^+^ T cell produces much more IL-17A protein than WT cells. In addition, TTP^–/–^ CD4^+^ T cells polarized under T_H_17 and T_H_1 conditions expressed more IL-17 and IL-6 mRNA than WT cells, whereas IFN-γ mRNA expression was no difference (Figure 3F and Supplementary Figures 4A,B). Compared to WT cells, the expression of the T_H_17 cell master transcription factor RORγt was decreased, Stat3 increased and Tbx21 mRNA kept no change in TTP^–/–^ CD4 T cells under either T_H_17 or T_H_1 skewing conditions (Figure 3F and Supplementary Figure 4B), further supporting that T_H_17 cell differentiation is not enhanced in CD4 T cells lacking TTP. Overall, these data indicate that the increased IL-17 secretion in TTP^–/–^ CD4^+^ T cells is due mainly to an enhanced synthesis of IL-17 at the single-cell level.

### TTP Controls the Effector Function of T_H_17 Cells by Reducing IL-17A mRNA Stability

**FIGURE 4:**
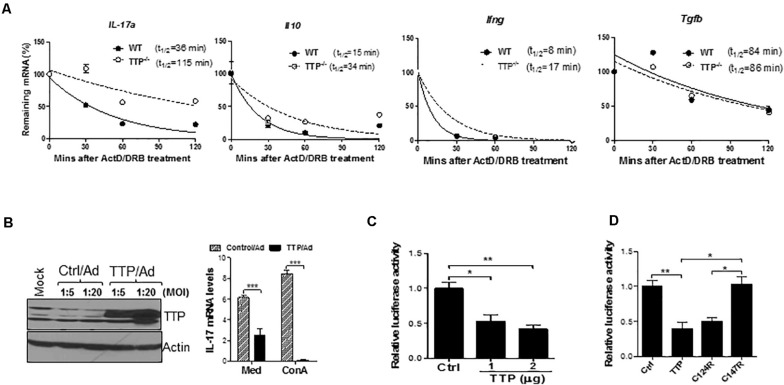
TTP controls effector function of T_H_17 cells by destabilizing IL-17 mRNA stability. **(A)** Splenic CD4^+^ T cells of *TTP*^– /–^ mice and wild-type (WT) littermates aged 6–8 months were stimulated under T_H_17 polarizing conditions for 4 days and then actinomycin D (ActD) (5 μg/mL) and DRB (10 μg/mL) were added. Total RNAs were extracted at 30, 60, 90, and 120 min after adding ActD and DRB. cDNAs were reverse-transcribed and residual cytokine as well as GAPDH mRNA were measured by real-time quantitative PCR (qPCR). The levels of residual cytokine mRNAs were normalized to GAPDH mRNA at each time point and half-life of the mRNA determined by comparing to the levels of mRNA before adding ActD and DRB. **(B)** Jurkat cells were infected with control adenovirus (Ctrl/Ad) or TTP-expressing adenovirus (TTP/Ad) at MOI = 5 and 20 for 24 h, followed by adding ConA (5 ug/mL) and then measuring IL-17 mRNA by qPCR and TTP protein by immunoblotting. **(C)** HEK293 cells were transiently cotransfected with IL-17A 3′ UTR–driven luciferase construct along with CMV–TTP vector, as well as empty vector (Ctrl), followed by measurement of luciferase activity in cell lysates after 40 h. **(D)** HEK293 cells were transiently cotransfected with IL-17A 3′ UTR–driven luciferase construct along with WT TTP, as well as two TTP zinc-finger mutant constructs (C124R and C147R), followed by measurement of luciferase activity in cell lysates after 40 h. Data shown as relative levels compared to luciferase activity in cells transfected with the empty vector (Ctrl) from three independent experiments. *p < 0.05, **p < 0.01, and ***p < 0.001 between groups.

To explore the mechanisms of TTP-mediated inhibition of IL-17A, we measured and compared IL-17 mRNA stability between WT and TTP^–/–^ T_H_17 cells. As shown in Figure 4A, the half-life of IL-17A mRNA was increased from 36 min in WT T_H_17 cells to 115 min in TTP^–/–^ T_H_17 cells. The half-lives of IL-10 and IFN-γ mRNAs were also increased in TTP^–/–^ T_H_17 cells compared to WT cells, whereas the TGF-β mRNA half-life remained similar (Figure 4A). In addition, the steady-state levels of IL-17A and TNF-α mRNA were also increased in TTP^–/–^ CD4 T cells in response to TCR and costimulatory signals (Supplementary Figure 5A). The mRNA half-lives of transcription factors important for T_H_17 cell development, such as IRF4 and IRF8, were actually decreased in TTP^–/–^ T_H_17 cells compared with WT cells (Supplementary Figures 5B,C), further indicating that TTP has minimal effects on T_H_17 cell differentiation. To further confirm the inhibitory effects of TTP on IL-17A, we introduced TTP by adenoviral transduction into Jurkat T cells and found that overexpression of TTP inhibited the expression of IL-17A mRNA (Figure 4B). To determine whether IL-17A 3′ UTR mediated the IL-17 inhibition, we cloned the 3′ UTR of IL-17A mRNA downstream of luciferase gene and cotransfected a TTP expression vector with the IL-17A–3′ UTR luciferase-reporter construct into HEK293 cells, followed by measuring luciferase activity. TTP inhibited IL-17A–3′ UTR–mediated luciferase activity (Figure 4C), indicating that TTP promotes IL-17A mRNA degradation through its 3′ UTR. TTP is a ZFP containing a CCCH tandem zinc-finger domain. Zinc fingers of this type have been found in many RNA-binding proteins and are responsible for binding to the 3′ UTRs of target mRNAs. To determine whether these zinc fingers were responsible for TTP inhibition of IL-17A secretion, we cotransfected WT TTP, or two TTP zinc-finger mutants (TTPΔC124R and TTPΔC147R), with the IL-17A-3′ UTR–luciferase-reporter plasmid into HEK293 cells, followed by measuring luciferase activity. As shown in Figure 4D, the C147R TTP mutant lost its suppressive effect on IL-17A-3′ UTR–regulated luciferase activity, whereas the C124R mutant retained luciferase suppressive activity. These data suggest that TTP via zinc finger promotes IL-17A mRNA degradation, resulting in inhibition of IL-17 secretion by CD4 T cells.

### T Cell-Specific TTP Conditional KO Mice Are Prone to DSS-Induced Intestinal Inflammation

**FIGURE 5:**
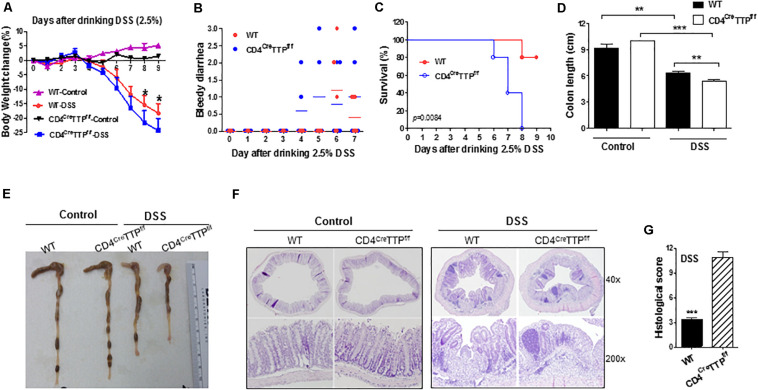
T cell-specific TTP conditional knockout mice are prone to DSS-induced colitis. Age- and body weight-matched CD4^Cre^TTP^f/f^ and control mice (8–12 weeks old) were administered with DSS in drinking water (2.5% wt/vol) for 5 days. Each group had five mice. Average body weight changes **(A)**, bloody diarrhea **(B)**, and survival **(C)** shown at indicated time points. On day 9, the lengths of colons of mice from treated groups were measured **(D)** and photographed **(E)**. Sections of colons in each group were stained with hematoxylin and eosin **(F)**. **(G)** Histological scores of colons collected from the above mice. Data are from four mice per group and represented as mean ± SD. *p < 0.05, **p < 0.01, and ***p < 0.001 between groups.

T_H_17 cells and IL-17 play important roles in intestinal inflammation (25, 26). DSS administration is commonly used to generate an acute mouse model of IBD. We used this model to determine the role of TTP in T_H_17-mediated intestinal inflammation. We fed WT and CD4^Cre^TTP^f/f^ mice DSS-containing water and found that the CD4^Cre^TTP^f/f^ mice displayed severe colitis compared with WT mice. Specifically, the CD4^Cre^TTP^f/f^ mice developed significantly greater weight loss and severe bloody diarrhea than the WT mice (Figures 5A,B). All CD4^Cre^TTP^f/f^ mice died due to severe colitis by 8 days after drinking DSS water, whereas 80% of WT mice survived (Figure 5C). Colon lengths were also greatly shortened in CD4^Cre^TTP^f/f^ mice compared with WT mice (Figures 5D,E). Histological analysis showed more severe tissue damage and more infiltrating inflammatory cells in the colons from DSS-treated CD4^Cre^TTP^f/f^ mice compared with WT mice (Figures 5F,G). These results indicate that T cell-specific TTP KO mice are vulnerable to DSS-induced colitis.

### IL-17 Mediates the DSS-Induced Colitis in T Cell-Specific TTP Conditional KO Mice

**FIGURE 6:**
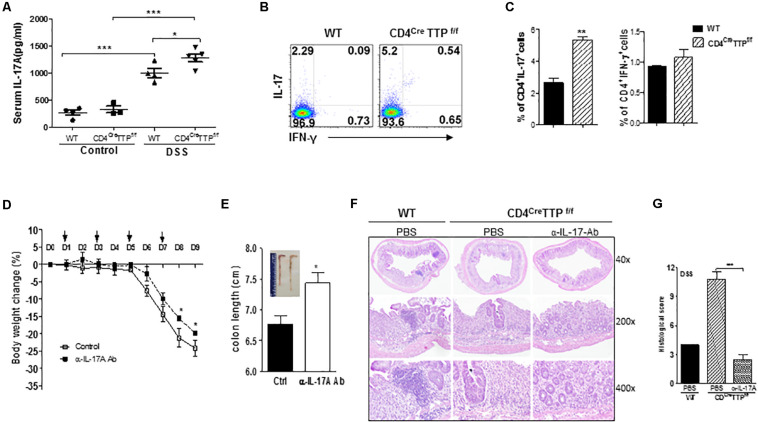
IL-17 mediates DSS-induced colitis in the T cell-specific TTP conditional knockout mice. Wild-type and CD4^Cre^TTP^f/f^ mice were fed 2.5% DSS water for 5 days and then switched to normal water for additional 4 days. Serum was isolated at day 9 and used to measure IL-17A levels by ELISA **(A)**. Lymphocytes isolated from mesenteric LN at day 9 were analyzed for intracellular cytokine expression by flow cytometry gated as CD4^+^ T cells **(B,C)**. CD4^Cre^TTP^f/f^ mice were received 150 mg of IL-17A–specific neutralizing mAb (*InVivo*MAb anti-mouse IL-17A, clone 17F3; BioXcell) intraperitoneally every other day starting on day 1 after DSS water administration. **(D)** Average body weight of the mice at indicated time points. **(E)** At day 9, colons were photographed (insert) and individual colon lengths were measured. **(F)** Sections of colons were analyzed with hematoxylin and eosin staining. **(G)** Histological scores of colons collected from the above mice. Data are from four or five mice per group and represented as mean ± SD. *p < 0.05, **p < 0.01, and ***p < 0.001 between groups.

To determine whether IL-17 is responsible for the DSS-induced colitis in this model, we measured IL-17 levels in serum of the CD4^Cre^TTP^f/f^ mice and control WT mice fed DSS-containing water. Serum levels of IL-17A were higher in both WT and CD4^Cre^TTP^f/f^ mice fed DSS water than in those without DSS, with even greater IL-17A levels in the CD4^Cre^TTP^f/f^ mice (Figure 6A). Next, we analyzed CD4 T-cell subsets and found a significant increase in IL-17A–producing CD4^+^ cells from the mesenteric LNs (MLNs) of CD4^Cre^TTP^f/f^ mice compared to WT mice, whereas the frequency of IFN-γ–producing CD4^+^ T cells was comparable (Figures 6B,C), further suggesting that IL-17 produced by the T_H_17 cells may contribute to the colitis in CD4^Cre^TTP^f/f^ mice. To confirm a causal relationship between IL-17 and colitis, we administered a neutralizing Ab specifically directed against IL-17A to CD4^Cre^TTP^f/f^ mice fed DSS-water and then closely monitored their body weights. The CD4^Cre^TTP^f/f^ mice receiving IL-17A neutralizing Ab showed significantly less body weight loss (Figure 6D) and had longer colons (Figure 6E) than the mice receiving control immunoglobulin G (IgG). Histological analysis also showed reduced intestinal inflammation and more intact structure in the colons of CD4^Cre^TTP^f/f^ mice given IL-17A neutralizing Ab (Figures 6F,G). These results demonstrate that IL-17 produced by T_H_17 cells contributes, at least partially, to the pathogenesis of DSS-induced colitis in CD4^Cre^TTP^f/f^ mice.

## Discussion

T_H_17 cells play important roles in chronic inflammation. Excessive T_H_17 cell development and IL-17 production are associated with the pathogenesis of several diseases, including autoimmune arthritis, multiple sclerosis, and IBD (31, 32). So far, most studies have focused on the development of T_H_17 cells and their transcriptional regulation by key transcription factors. Relatively little is known about how T_H_17 cells are regulated at the post-transcriptional level. In this study, we have demonstrated that the RNA-binding protein TTP negatively regulates T_H_17 cell function at the post-transcriptional level by enhancing degradation of the IL-17 mRNA. It has been reported that conventional TTP^–/–^ mice develop a severe inflammatory syndrome with elevated levels of circulating inflammatory cytokines due to increased mRNA stability (22, 27, 28, 33–40), demonstrating that TTP is an important regulator for control of inflammatory responses. In order to evaluate the role of TTP in regulation of T-cell development and function, we generated CD4-specifc TTP conditional KO mice. The CD4^Cre^TTP^f/f^ mice exhibited different phenotypes from conventional TTP^–/–^ mice. One obvious difference was that the CD4^Cre^TTP^f/f^ mice appeared to undergo normal growth, whereas the conventional TTP^–/–^ mice had retarded growth (Figure 1A). Another difference in the CD4^Cre^TTP^f/f^ mice was the lack of the early onset and severe inflammatory arthritis universally seen in the conventional TTP^–/–^ mice (26). Although younger CD4^Cre^TTP^f/f^ mice appeared normal, they did develop atopic dermatitis and had elevated skin T_H_17 cells and serum levels of IL-17A when they aged past 5–8 months. Frasca et al. (41) showed that TTP levels were higher in activated B cells from old mice as compared with young mice. These data suggest that TTP plays a role in controlling lymphocyte activation and chronic inflammation during the aging processes.

The spontaneous chronic inflammation in conventional TTP^–/–^ mice is considered to be at least partly TNF-α dependent, as TNF mRNA was stabilized in TTP^–/–^ macrophages after stimulation with lipopolysaccharide (16, 30), and treatment of the conventional TTP KO mice with Abs to TNF-α (26), or breeding to TNF-α receptor–deficient mice (15), prevented development of the inflammatory syndrome. However, specific deletion of TTP in myeloid cells did not recapitulate the phenotypes of conventional TTP^–/–^ mice (30), indicating the involvement of other cell types in the development of the inflammatory syndromes. Indeed, we found that both CD4^+^ and CD8^+^ T cells (Supplementary Figure 6) in the T cell-specific TTP-deficient mice produced much higher levels of IL-17A compared with the cells of WT mice. The increased IL-17A along with spontaneous dermatitis development in old CD4^Cre^TTP^f/f^ mice demonstrates a role for TTP in regulating T cell-mediated inflammation. Studies from our laboratory and others show that TTP targets IL-23 and IL-6 mRNA for degradation (27, 34, 36, 37). Because both cytokines are important for T_H_17 cell differentiation, these collective results suggest that TTP may affect the development of T_H_17 cells. Surprisingly, several key transcription factors important for T_H_17 cell differentiation, such as RORγt, IRF4, IRF8, were not increased in TTP^–/–^ T_H_17 cells, indicating that TTP may affect T_H_17 cell function rather than T_H_17 cell development. Indeed, TTP^–/–^ T_H_17 cells produced more IL-17A than WT T_H_17 cells at the single-cell level (Figures 3A–E), and the IL-17 mRNA stability was increased in TTP^–/–^ T_H_17 cells (Figure 4A). Our results indicate that TTP negatively regulates T_H_17 cell function by promoting mRNA degradation of its signature cytokine IL-17. IFN-γ might also play a role in the development of skin inflammation in aged CD4^Cre^TTP^f/f^ mice, as the levels of IFN-γ were also increased in the T_H_0 and T_H_17 cells lacking TTP (Figure 3A), which is consistent with a previous report that TTP promotes IFN-γ mRNA degradation (35). Molle et al. (28) reported that IL-17 is involved in the generation of joint inflammation in conventional TTP^–/–^ mice, as deletion of IL-17 in TTP^–/–^ mice ameliorates the inflammation. They also reported that the differentiation of T_H_17 cells from naive CD4 T cells lacking TTP was normal (28), which is in line with our observation. These data together suggest that TTP regulates T_H_17 cell function by targeting IL-17 mRNA stability, without directly affecting T_H_17 cell differentiation.

Signaling through many TLRs and other environmental stimuli can induce TTP expression in macrophages, which in turn prevents excessive cytokine production and inflammation. TTP suppresses the expression of proinflammatory cytokines by binding directly to AREs in the 3′ UTR of their mRNAs, leading to their deadenylation and decay. In primary CD4 T cells, TTP inhibits IL-17 expression by directly promoting mRNA degradation via the IL-17 3′ UTR (Figures 4A,C). Using human T-cell line HuT102 and Jurkat cells, Lee et al. (42) reported that TTP bound to several AREs in the 3′ UTR of IL-17 mRNA for degradation of human IL-17 transcript. These data suggest that TTP regulates the expression of macrophage cytokines and T-cell cytokines through similar mechanisms. It is known that TTP regulates targeted gene expression through several mechanisms, such as mRNA localization or translation efficiency or other RNA modifications in addition to mRNA stabilization (25, 43). These mechanisms may also contribute to IL-17 regulation by TTP in T cells.

IBDs in human are characterized by chronic intestinal inflammation mediated by several factors. Both protective and pathogenic functions of IL-17 have been reported in different experimental models of colitis. Adoptive transfer of IL-17^–/–^ CD45RB^hi^ T cells into RAG^–/–^ recipient mice induced a more severe wasting disease compared with WT counterparts (44). In contrast, IL-17 deficiency resulted in resistance to dextran sulfate sodium–induced colitis in mice, indicating a pathogenic role of IL-17 in intestinal inflammation (12). Our data show increased IL-17 in CD4^Cre^TTP^f/f^ mice, along with accelerated wasting disease compared with WT littermates. It has been reported that neutralizing IL-17 activity enhanced the development of DSS-colitis in WT mice (45). Therefore, we focused our study on whether IL-17 in the CD4 conditional TTP KO mice played a role in DSS-induced colitis by administering IL-17 neutralization Ab only to KO mice. As shown in Figure 6D, neutralizing IL-17 reduced the severity of wasting disease, supporting a pathogenic role for IL-17 in intestinal inflammation observed in the CD4^Cre^TTP^f/f^ mice. Notably, although neutralization of IL-17 reduced DSS-induced colitis, mice still developed significant weight loss. This suggests that other factors regulated by TTP may also contribute to DSS-colitis in the T cell-specific TTP conditional KO mice. Although CD4cre is broadly used to generating conditional gene targeting in CD4 T cells, several types of cells express CD4, including thymic CD8, CD4-expressing DCs, and monocytes. Therefore, the phenotypes observed in the CD4^Cre^TTP^f/f^ mice may be mediated by several types of cells. TNF-α is known to be important in the pathogenesis of intestinal inflammation. TNF-α expression was indeed higher in TTP^–/–^ T_H_17 cells than WT T_H_17 cells (Supplementary Figure 4B). TNF expression in TTP^–/–^T_H_17 cells may also contribute to the DSS-induced colitis in the CD4^Cre^TTP^f/f^ mice. IL-6 could be involved, because TTP deficiency also led to increased IL-6 in the serum of CD4 T cell-specific TTP KO mice (Figure 3F and Supplementary Figure 4A). More study is needed to further investigate the roles of IL-6 and other players in the pathogenesis of intestinal inflammation mediated by TTP. T_H_17 cell-produced GM-CSF plays an essential role in autoimmune inflammation (such as encephalitis) mediated mainly by a positive feedback loop between GM-CSF secreted by T_H_17 cells and the production of IL-23 by antigen-presenting cells (46, 47). We recently report that GM-CSF–producing CD4 T cells may be a unique subset of T helper cells, namely, T_H_-GF-CSF, important for T cell-mediated inflammation (48). GM-CSF has been reported to mediate intestine inflammation (49). Considering TTP mediates GM-CSF expression (20), it is possible that GM-CSF may also contribute to the phenotypes in the CD4^Cre^TTP^f/f^ mice.

In summary, our results reveal a new mechanism for regulating T_H_17 function and T_H_17-mediated inflammation, through post-transcriptional regulation of IL-17 mRNA decay by TTP, suggesting that TTP might be a novel therapeutic target for treatment of the T_H_17-mediated diseases.

## Materials and Methods

### Mice

CD4^Cre^TTP^f/f^ mice were obtained by crossing mice expressing Cre recombinase under the control of the murine CD4 promoter (CD4^Cre^) mice purchased from the Jackson Laboratory (Bar Harbor, ME, United States) (29) with the previously described TTP^flox/flox^ mice (30). All mice were on the C57BL/6 background and were bred at the animal facility of Saint Louis University. Animal experiments were approved by the Institutional Animal Care and Use Committee at Saint Louis University and were performed according to federal and institutional guidelines.

### Cell Lines

Jurkat cells were purchased from ATCC and cultured in RPMI-1640 with 10% (vol/vol) fetal calf serum (Sigma, St. Louis, MO, United States; endotoxin NMT 10.0 EU/mL) supplemented with 2 mM glutamine and 100 units/mL of penicillin and streptomycin (Sigma). HEK293T cells were purchased from ATCC and cultured in Dulbecco modified eagle medium (4.5 g/L) with 10% (vol/vol) fetal calf serum and 100 units/mL of penicillin and streptomycin purchased from Sigma as described above.

### Antibodies and Flow Cytometry

The following Abs were purchased from BD Biosciences: anti-CD3-APC (clone 145-2C11), anti–CD4-PE (clone H129.19), anti–CD8-PE (clone 53-6.7), anti–CD4-PerCP (clone RM4-5), anti–CD44-FITC (clone IM7). For intracellular staining of cytokines, cells were stimulated for 4 h with PMA (phorbol 12-myristate 13-acetate; 50 ng/mL; Sigma) and ionomycin (0.5 μg/mL; Sigma) in the presence of Monensin (GolgiStop; 0.7 μL/mL; BD Biosciences) for 4 h and then fixed and permeabilized for intracellular cytokine testing according to the manufacturer’s instructions (BD Biosciences). Antibodies used were as follows: anti-IFN-γ–APC (clone XMG1.2), anti–IL-17A–PE (clone TC11-18H10.1), anti–IL-17A–AF488 (clone TC11-18H10.1), anti-IFN-γ–AF700 (clone XMG1.2), from BD Biosciences. Cells were analyzed on a FACSCanto II or FACS LSR II (BD), and data were analyzed with FlowJo software (TreeStar).

### T-Cell Stimulation and Differentiation

CD4^+^ T cells, purified using the DynaBeads FlowComp mouse CD4 kit (Invitrogen) or EasySep Mouse Naive CD4^+^ T Cell Iso Kit (Stemcell), were activated by plate-coated anti-CD3 (1 μg/mL; 145-2C11; Biolegend) and anti-CD28 (1 μg/mL; 37.51; Biolegend) Abs for 72 h in complete cell culture medium (RPMI 1640) supplemented with 10% heat-inactivated fetal calf serum, 2 mM L-glutamine, penicillin–streptomycin, non-essential amino acids, sodium pyruvate, 10 mM HEPES, and 50 μM 2-mercaptoethanol. Supernatants were collected for measuring cytokines by enzyme-linked immunosorbent assay (ELISA) (BD Bioscience). For *in vitro* T-cell differentiation, CD4^+^ T cells were activated with plate-coated anti-CD3 (2 μg/mL) and anti-CD28 (2 μg/mL) Abs under the following conditions: for T_H_1: anti-IL-4 20 μg/mL (11B11; BD), IL-12 10 U/mL (Peprotech); for T_H_17: IL-6 50 ng/mL (Peprotech), TGF-β 1 ng/mL (Peprotech), IL-23 20 ng/mL (Peprotech), anti-IL-4 10 μg/mL (11B11; BD), and anti-IFN-γ 10 μg/mL (XMG1.2; BD). Cells were cultured for 4 days before activated with PMA (50 ng/mL; Sigma) and ionomycin (0.5 μg/mL; Sigma) for 4 h for analysis of cytokines and surface markers with flow cytometry.

### Plasmid Constructs

Full-length 3′ UTR of murine IL-17A was retrieved from the website AREsite (Universitat Wien). A 656-bp fragment of the mouse IL-17A 3′ UTR amplified from cDNA of mouse T cells was cloned downstream of the luciferase gene in pGL3 control vector (Promega) between *Xba*I and *Fse*I sites. Luciferase-reporter gene expression is driven by SV40 promoter and has been used by us and others to study mRNA stability controlled by downstream inserted 3′ UTR or siRNA (27, 50–54). WT TTP and two mutant TTP plasmids were generated in Perry Blackshear’s laboratory (55).

### Luciferase Assay

Transient transfections were performed by electroporation. Briefly, for each condition 0.4 mL of HEK293T-cell suspension containing 1 × 10^7^ cells was mixed with 3 μg of total DNA (including reporter, effector, internal control, and carrier DNA) and electroporated in 0.45-cm electroporation cuvettes (Gene Pulser II; Bio-Rad Laboratories, Hercules, CA, United States) at 975 microfarade and 300 V in RPMI 1640 medium without serum. The transfected cells from different cuvettes were resuspended in RPMI 1640 containing 10% fetal bovine serum (FBS), 2 mM glutamine, 10 μM chloroquine, and antibiotics and were added to 24-well plates and incubated for 40 h prior to harvesting. To measure luciferase activity, cells were pelleted by centrifugation and resuspended in 100 μL of lysis buffer containing 125 mM Tris-phosphate pH 7.8; 10 mM DTT; 10 mM 1-2-diaminocyclohexane-tetraacetic acid; 50% glycerol; and 5% Triton-X100. Luciferase activity was measured in cell lysates with Luciferase Reporter Assay system (Promega). Transfection efficiency was routinely monitored by β-galactosidase (β-gal) assay by cotransfection with 3 μg of pCMV-β-gal plasmid. Variability in β-gal activity between samples was typically within 5%. Lysates were used for both luciferase and β-gal assays.

### Quantitative Reverse Transcription–Polymerase Chain Reaction

Total RNA was extracted using TRIzol (Invitrogen, Life Technologies). Reverse transcription (RT) reactions were carried out as follows: 1-μg aliquots of total RNA were mixed with 1 μL oligo dT primers (0.5 mg/mL), 1 μL 10 mM dNTPs, and ddH_2_O to equalize volumes of all samples at 12 μL. The mixture was heated at 65°C for 5 min, quenched on ice, spun down briefly, and 8 μL of a Master Mix was added. The RT Master Mix consisted of 4 μL 5 × first-strand buffer (Invitrogen), 2 μL 0.1 M DTT, 1 μL RNase inhibitor (40 U/μL; Invitrogen), and 1 μL Superscript II (200 μL/μL, Invitrogen). The reaction was incubated at 42°C for 60 min and then at 70°C for 15 min, followed by 4°C soak. To each sample (in 20 μL total volume), 80 μL ddH_2_O was added; 5 μL diluted cDNA was used for each polymerase chain reaction (PCR) reaction of 25 μL volume. PCR was performed using two-step qRT-PCR with SYBR green (Invitrogen, Life Technologies) with ABI Prism 7700 Sequence detector/Real Time PCR machine. Results were analyzed using ΔΔCT method with glyceraldehyde-3-phosphate dehydrogenase (GAPDH) as an endogenous reference control. Primers for specific murine targets were as follows: *Ifn-*γ, forward, 5′-CCATCCTTTTGCCAGTTCCTC-3′, and reverse, 5′-ATGAACGCTACAC ACTGCATC-3′; *Il17a*, forward, 5′-CTCCAGAAGGCCCTCAGACTAC-3′, and reverse, 5′-GG GTCTTCATTGCGGTGG-3′; *Tgfb*, forward, 5′-TAAGAGGTC ACCCGCGTGCT-3′, and reverse, 5′-AAAGACAGCCACTC AGGCGTA-3′; Rorc, forward, 5′-AAGATCTGCAGCTTT TCCACA-3′, and reverse, 5′-TTTGGAACTGGCTTTCCATC-3′; *Stat3*, forward, 5′-CAGACTGGTTGTTTCCATTCAGAT-3′, and reverse, 5′-ACCCAACAGCCGCCGTAG-3′; *Tbx21*, forward, 5′-ATGCGTACATGGACTCAAAGTT-3″, and reverse, 5′-TTT CCAA GAGACCCAGTTCATTG-3′; *Il6*, forward, 5′-GGAAATT GGGGTAGGAAGGA-3′, and reverse, 5′-TGTGCAATGGCAA TTCTGAT-3′; GAPDH, forward, 5′-TGGCCTACATGGCCT CCA-3′, and reverse, 5′-TCCCTAGGCCCCTCCTGTTAT-3′.

### mRNA Stability

CD4^+^ T cells were cultured under T_H_17 polarization conditions before adding actinomycin D (5 μg/mL; Sigma) and 5,6-dichlorobenzimidazole riboside (DRB) (10 μg/mL; Sigma), and then total RNAs collected at different times. cDNAs were used to measure the remaining mRNA for calculating the half-life of each mRNA as described previously (27, 56, 57). In brief, the half-lives of mRNAs were calculated separately in WT and TTP KO cells and then compared. The remaining levels of mRNAs at 30, 60, 90, and 120 min after blocking *de novo* RNA synthesis with ActD/DRB were first normalized to housekeeping GAPDH at each time point and then calculated against the levels of mRNAs at *t* = 0, which was set as 100%.

### Enzyme-Linked Immunosorbent Assays

Serum from blood of WT and KO mice treated with or without DSS, as well as supernatants from CD4 T cells, was harvested at described in the text and stored at −70°C. Mouse IL-17 was detected using ELISA MAX^TM^ Deluxe Set Mouse IL-17A kit (Biolegend) according to the manufacturer’s instructions. Concentrations were calculated by regression analysis of a standard curve.

### Western Blotting

Cells were lysed in RIPA buffer for 30 min on ice. Protein lysates (25 μg) were loaded on a 10% sodium dodecyl sulfate–polyacrylamide gel electrophoresis. Gels were transferred to nitrocellulose membrane and blocked in 5% non-fat milk in Tris buffer, pH 8.0, at room temperature for 2 h. The membrane was blotted with anti-TTP Primary Ab (clone 3A2; 1 mg/mL; Santa Cruz Biotechnology) in Tris buffer containing 5% milk powder and left overnight at 4°C. After extensive washing, blots were subjected to horseradish peroxidase–conjugated sheep anti-mouse Ig secondary Ab at a 1:5,000 dilution in 5% milk and then detected with enhanced chemiluminescence detection (PerkinElmer Life Sciences Inc., Boston, MA, United States).

### DSS Colitis Model

Colitis was induced in mice with drinking water containing 2.5% DSS (MP Biomedicals) for 5 days and then replaced with normal water. Mice were monitored for weight changes, diarrhea, bloody stools, and overall health. Mice were removed from the study when their body weight loss exceeded 25% of their original body weight and counted as death.

### Histologic Colitis Assessment and Colon Length

The entire colon was removed from cecum to anus, measured, and fixed in 10% neutral-buffered formalin. Five-micrometer-thick sections were stained with hematoxylin and eosin for microscopic examination. Histological scores were assigned by a pathologist blinded to the experimental groups based on the extent and severity of inflammation, ulceration, and hyperplasia of the mucosa. The final score was calculated as the sum of individual factors, multiplied by the extent of tissue involvement. Severity scores for inflammation were as follows: 0 = normal (within normal limits), 1 = mild (small, focal, or widely separated, limited to lamina propria), 2 = moderate (multifocal or locally extensive, extending to submucosa), and 3 = severe (transmural inflammation with ulcers covering >20 crypts). Multiplied factors as extent of lesions were as follows: 0 = normal (0% involvement), 1 = up to 25% involvement, 2 = 25–50% involvement, 3 = over 51–50% involvement, and 4 = over 76–100% involvement. Spleen and MLNs were mechanically dissociated, and red cells were lysed in ACK lysis buffer. Cell suspensions were washed, enumerated, and stored in RPMI 1640 containing 10% FBS (Cellgro, Herndon, VA, United States) on ice until used.

### Statistical Analysis

Data were analyzed with Prism software 5.0 (GraphPad). For standard data sets, data were shown as mean ± SD, and an unpaired two-tailed Student’s *t*-test was used. For multiple groups, one-way analysis of variance was used. ^∗^*p* < 0.05, ^∗∗^*p* < 0.01, ^∗∗∗^*p* < 0.001 between indicated groups.

## Data Availability Statement

The raw data supporting the conclusions of this article will be made available by the authors, without undue reservation.

## Ethics Statement

The animal study was reviewed and approved by Institutional Animal Care and Use Committee at the Saint Louis University.

## Author Contributions

JLi contributed to the concept and design and study supervision. JLi, HN, and HP contributed to the development of the methodology. DS, PB, HN, HP, and QW contributed to acquisition of the data (provided animals, plasmids and reagents, etc.). JL, HN, JLa, LW, and HP contributed to analysis and interpretation of the data (e.g., statistical analysis, biostatistics, and computational analysis). JL, HP, DH, MF, and RH contributed to writing, review, and revision of the manuscript. PB contributed to administrative, technical, and material support (reporting or organizing data and construction database). All authors contributed to the article and approved the submitted version.

## Conflict of Interest

The authors declare that the research was conducted in the absence of any commercial or financial relationships that could be construed as a potential conflict of interest.

## References

[1] Korn T Bettelli E Oukka M Kuchroo VK. IL-17 and Th17 Cells. *Annu Rev Immunol.* (2009) 27:485–517. 19132915 10.1146/annurev.immunol.021908.132710

[2] Weaver CT Hatton RD Mangan PR Harrington LE. IL-17 family cytokines and the expanding diversity of effector T cell lineages. *Annu Rev Immunol.* (2007) 25:821–52. 17201677 10.1146/annurev.immunol.25.022106.141557

[3] Yang XP Ghoreschi K Steward-Tharp SM Rodriguez-Canales J Zhu J Grainger JR Opposing regulation of the locus encoding IL-17 through direct, reciprocal actions of STAT3 and STAT5. *Nat Immunol.* (2011) 12: 247–54. 21278738 10.1038/ni.1995 PMC3182404

[4] Mangan PR Harrington LE O’Quinn DB Helms WS Bullard DC Elson CO Transforming growth factor-beta induces development of the T(H)17 lineage. *Nature.* (2006) 441:231–4. 16648837 10.1038/nature04754

[5] Ivanov II McKenzie BS Zhou L Tadokoro CE Lepelley A Lafaille JJ The orphan nuclear receptor RORgammat directs the differentiation program of proinflammatory IL-17+ T helper cells. *Cell.* (2006) 126: 1121–33. 16990136 10.1016/j.cell.2006.07.035

[6] Park H Li Z Yang XO Chang SH Nurieva R Wang YH A distinct lineage of CD4 T cells regulates tissue inflammation by producing interleukin 17. *Nat Immunol.* (2005) 6:1133–41. 16200068 10.1038/ni1261 PMC1618871

[7] Harrington LE Hatton RD Mangan PR Turner H Murphy TL Murphy KM Interleukin 17-producing CD4+ effector T cells develop via a lineage distinct from the T helper type 1 and 2 lineages. *Nat Immunol.* (2005) 6:1123–32. 16200070 10.1038/ni1254

[8] Langrish CL Chen Y Blumenschein WM Mattson J Basham B Sedgwick JD IL-23 drives a pathogenic T cell population that induces autoimmune inflammation. *J Exp Med.* (2005) 201: 233–40. 15657292 10.1084/jem.20041257 PMC2212798

[9] Marinoni B Ceribelli A Massarotti MS Selmi C. The Th17 axis in psoriatic disease: pathogenetic and therapeutic implications. *Auto Immun Highlights.* (2014) 5:9–19. 10.1007/s13317-013-0057-4 26000152 PMC4389010

[10] Kugyelka R Kohl Z Olasz K Mikecz K Rauch TA Glant TT Enigma of IL-17 and Th17 cells in rheumatoid arthritis and in autoimmune animal models of arthritis. *Mediators Inflamm.* (2016) 2016:6145810. 10.1155/2016/6145810 PMC4745575 26903711

[11] Sarra M Pallone F Macdonald TT Monteleone G. IL-23/IL-17 axis in IBD. *Inflamm Bowel Dis.* (2010) 16:1808–13. 20222127 10.1002/ibd.21248

[12] Ito R Kita M Shin-Ya M Kishida T Urano A Takada R Involvement of IL-17A in the pathogenesis of DSS-induced colitis in mice. *Biochem Biophys Res Commun.* (2008) 377:12–6. 18796297 10.1016/j.bbrc.2008.09.019

[13] Iwakura Y Nakae S Saijo S Ishigame H. The roles of IL-17A in inflammatory immune responses and host defense against pathogens. *Immunol Rev.* (2008) 226:57–79. 10.1111/j.1600-065x.2008.00699.x 19161416

[14] Blackshear PJ. Tristetraprolin and other CCCH tandem zinc-finger proteins in the regulation of mRNA turnover. *Biochem Soc Trans.* (2002) 30:945–52. 12440952 10.1042/bst0300945

[15] Carballo E Blackshear PJ. Roles of tumor necrosis factor-alpha receptor subtypes in the pathogenesis of the tristetraprolin-deficiency syndrome. *Blood.* (2001) 98:2389–95. 11588035 10.1182/blood.v98.8.2389

[16] Carballo E Lai WS Blackshear PJ. Feedback inhibition of macrophage tumor necrosis factor-alpha production by tristetraprolin. *Science.* (1998) 281:1001–5. 9703499 10.1126/science.281.5379.1001

[17] Qiu LQ Lai WS Bradbury A Zeldin DC Blackshear PJ. Tristetraprolin (TTP) coordinately regulates primary and secondary cellular responses to proinflammatory stimuli. *J Leukoc Biol.* (2015) 97:723–36. 25657290 10.1189/jlb.3A0214-106R PMC4370050

[18] Sauer I Schaljo B Vogl C Gattermeier I Kolbe T Muller M Interferons limit inflammatory responses by induction of tristetraprolin. *Blood.* (2006) 107:4790–7. 16514065 10.1182/blood-2005-07-3058 PMC3963709

[19] Lai WS Carballo E Strum JR Kennington EA Phillips RS Blackshear PJ. Evidence that tristetraprolin binds to AU-rich elements and promotes the deadenylation and destabilization of tumor necrosis factor alpha mRNA. *Mol Cell Biol.* (1999) 19:4311–23. 10330172 10.1128/mcb.19.6.4311 PMC104391

[20] Carballo E Lai WS Blackshear PJ. Evidence that tristetraprolin is a physiological regulator of granulocyte-macrophage colony-stimulating factor messenger RNA deadenylation and stability. *Blood.* (2000) 95:1891–9. 10706852

[21] Sawaoka H Dixon DA Oates JA Boutaud O. Tristetraprolin binds to the 3′-untranslated region of cyclooxygenase-2 mRNA. A polyadenylation variant in a cancer cell line lacks the binding site. *J Biol Chem.* (2003) 278: 13928–35. 12578839 10.1074/jbc.M300016200

[22] Ogilvie RL Abelson M Hau HH Vlasova I Blackshear PJ Bohjanen PR. Tristetraprolin down-regulates IL-2 gene expression through AU-rich element-mediated mRNA decay. *J Immunol.* (2005) 174:953–61. 15634918 10.4049/jimmunol.174.2.953

[23] Stoecklin G Tenenbaum SA Mayo T Chittur SV George AD Baroni TE Genome-wide analysis identifies interleukin-10 mRNA as target of tristetraprolin. *J Biol Chem.* (2008) 283:11689–99. 18256032 10.1074/jbc.M709657200 PMC2431067

[24] Datta S Biswas R Novotny M Pavicic PG Jr. Herjan T Mandal P Tristetraprolin regulates CXCL1 (KC) mRNA stability. *J Immunol.* (2008) 180:2545–52. 18250465 10.4049/jimmunol.180.4.2545

[25] Brooks SA Blackshear PJ. Tristetraprolin (TTP): interactions with mRNA and proteins, and current thoughts on mechanisms of action. *Biochim Biophys Acta.* (2013) 1829:666–79. 23428348 10.1016/j.bbagrm.2013.02.003 PMC3752887

[26] Taylor GA Carballo E Lee DM Lai WS Thompson MJ Patel DD A pathogenetic role for TNF alpha in the syndrome of cachexia, arthritis, and autoimmunity resulting from tristetraprolin (TTP) deficiency. *Immunity.* (1996) 4:445–54. 8630730 10.1016/s1074-7613(00)80411-2

[27] Qian X Ning H Zhang J Hoft DF Stumpo DJ Blackshear PJ Posttranscriptional regulation of IL-23 expression by IFN-gamma through tristetraprolin. *J Immunol.* (2011) 186:6454–64. 21515794 10.4049/jimmunol.1002672 PMC3914637

[28] Molle C Zhang T Ysebrant de Lendonck L Gueydan C Andrianne M Sherer F Tristetraprolin regulation of interleukin 23 mRNA stability prevents a spontaneous inflammatory disease. *J Exp Med.* (2013) 210:1675–84. 23940256 10.1084/jem.20120707 PMC3754859

[29] Lee PP Fitzpatrick DR Beard C Jessup HK Lehar S Makar KW A critical role for Dnmt1 and DNA methylation in T cell development, function, and survival. *Immunity.* (2001) 15:763–74. 11728338 10.1016/s1074-7613(01)00227-8

[30] Qiu LQ Stumpo DJ Blackshear PJ. Myeloid-specific tristetraprolin deficiency in mice results in extreme lipopolysaccharide sensitivity in an otherwise minimal phenotype. *J Immunol.* (2012) 188:5150–9. 22491258 10.4049/jimmunol.1103700 PMC3345041

[31] Aggarwal S Gurney AL. IL-17: prototype member of an emerging cytokine family. *J Leukoc Biol.* (2002) 71:1–8. 11781375

[32] Moseley TA Haudenschild DR Rose L Reddi AH. Interleukin-17 family and IL-17 receptors. *Cytokine Growth Factor Rev.* (2003) 14:155–74. 12651226 10.1016/s1359-6101(03)00002-9

[33] Jalonen U Nieminen R Vuolteenaho K Kankaanranta H Moilanen E. Down-regulation of tristetraprolin expression results in enhanced IL-12 and MIP-2 production and reduced MIP-3alpha synthesis in activated macrophages. *Mediators Inflamm.* (2006) 2006:40691. 10.1155/MI/2006/40691 PMC1775030 17392586

[34] Lee HH Yang SS Vo MT Cho WJ Lee BJ Leem SH Tristetraprolin down-regulates IL-23 expression in colon cancer cells. *Mol Cells.* (2013) 36:571–6. 24292977 10.1007/s10059-013-0268-6 PMC3887959

[35] Ogilvie RL Sternjohn JR Rattenbacher B I Vlasova A Williams DA Hau HH Tristetraprolin mediates interferon-gamma mRNA decay. *J Biol Chem.* (2009) 284:11216–23. 19258311 10.1074/jbc.M901229200 PMC2670126

[36] Van Tubergen E Vander Broek R Lee J Wolf G Carey T Bradford C Tristetraprolin regulates interleukin-6, which is correlated with tumor progression in patients with head and neck squamous cell carcinoma. *Cancer.* (2011) 117:2677–89. 21656745 10.1002/cncr.25859 PMC3574798

[37] Zhao W Liu M D’Silva NJ Kirkwood KL. Tristetraprolin regulates interleukin-6 expression through p38 MAPK-dependent affinity changes with mRNA 3′ untranslated region. *J Interferon Cytokine Res.* (2011) 31:629–37. 21457063 10.1089/jir.2010.0154 PMC3151618

[38] Zhu W Brauchle MA Di Padova F Gram H New L Ono K Gene suppression by tristetraprolin and release by the p38 pathway. *Am J Physiol Lung Cell Mol Physiol.* (2001) 281:L499–508. 11435226 10.1152/ajplung.2001.281.2.L499

[39] Deleault KM Skinner SJ Brooks SA. Tristetraprolin regulates TNF TNF-alpha mRNA stability via a proteasome dependent mechanism involving the combined action of the ERK and p38 pathways. *Mol Immunol.* (2008) 45:13–24. 10.1016/j.molimm.2007.05.017 17606294

[40] Fabris M Tolusso B Di Poi E Tomietto P Sacco S Gremese E Mononuclear cell response to lipopolysaccharide in patients with rheumatoid arthritis: relationship with tristetraprolin expression. *J Rheumatol.* (2005) 32:998–1005. 15940758

[41] Frasca D Landin AM Alvarez JP Blackshear PJ Riley RL Blomberg BB. Tristetraprolin, a negative regulator of mRNA stability, is increased in old B cells and is involved in the degradation of E47 mRNA. *J Immunol.* (2007) 179:918–27. 17617583 10.4049/jimmunol.179.2.918

[42] Lee HH Yoon NA Vo MT Kim CW Woo JM Cha HJ Tristetraprolin down-regulates IL-17 through mRNA destabilization. *FEBS Lett.* (2012) 586:41–6. 22138182 10.1016/j.febslet.2011.11.021

[43] Brook M Tchen CR Santalucia T McIlrath J Arthur JS Saklatvala J Posttranslational regulation of tristetraprolin subcellular localization and protein stability by p38 mitogen-activated protein kinase and extracellular signal-regulated kinase pathways. *Mol Cell Biol.* (2006) 26: 2408–18. 16508015 10.1128/MCB.26.6.2408-2418.2006 PMC1430283

[44] O’Connor W Jr. Kamanaka M Booth CJ Town T Nakae S Iwakura Y A protective function for interleukin 17A in T cell-mediated intestinal inflammation. *Nat Immunol.* (2009) 10:603–9. 19448631 10.1038/ni.1736 PMC2709990

[45] Ogawa A Andoh A Araki Y Bamba T Fujiyama Y. Neutralization of interleukin-17 aggravates dextran sulfate sodium-induced colitis in mice. *Clin Immunol.* (2004) 110:55–62. 10.1016/j.clim.2003.09.013 14962796

[46] McGeachy MJ. GM-CSF: the secret weapon in the T(H)17 arsenal. *Nat Immunol.* (2011) 12:521–2. 21587311 10.1038/ni.2044

[47] Codarri L Gyulveszi G Tosevski V Hesske L Fontana A Magnenat L RORgammat drives production of the cytokine GM-CSF in helper T cells, which is essential for the effector phase of autoimmune neuroinflammation. *Nat Immunol.* (2011) 12:560–7. 21516112 10.1038/ni.2027

[48] Hu Y Xu F Zhang R Legarda D Dai J Wang D Interleukin-1beta-induced IRAK1 ubiquitination is required for TH-GM-CSF cell differentiation in T cell-mediated inflammation. *J Autoimmun.* (2019) 102:50–64. 10.1016/j.jaut.2019.04.010 31080014

[49] Griseri T I Arnold C Pearson C Krausgruber T Schiering C Franchini F Granulocyte macrophage colony-stimulating factor-activated eosinophils promote interleukin-23 driven chronic colitis. *Immunity.* (2015) 43:187–99. 26200014 10.1016/j.immuni.2015.07.008 PMC4518500

[50] Greene SB Gunaratne PH Hammond SM Rosen JM. A putative role for microRNA-205 in mammary epithelial cell progenitors. *J Cell Sci.* (2010) 123:606–18. 20103531 10.1242/jcs.056812 PMC2818197

[51] Jin Y Chen Z Liu X Zhou X. Evaluating the microRNA targeting sites by luciferase reporter gene assay. *Methods Mol Biol.* (2013) 936:117–27. 23007504 10.1007/978-1-62703-083-0_10 PMC3646406

[52] Chowdhury B Krishnan S Tsokos CG Robertson JW Fisher CU Nambiar MP Stability and translation of TCR zeta mRNA are regulated by the adenosine-uridine-rich elements in splice-deleted 3′ untranslated region of zeta-chain. *J Immunol.* (2006) 177:8248–57. 17114503 10.4049/jimmunol.177.11.8248

[53] He G Sun D Ou Z Ding A. The protein Zfand5 binds and stabilizes mRNAs with AU-rich elements in their 3′-untranslated regions. *J Biol Chem.* (2012) 287:24967–77. 22665488 10.1074/jbc.M112.362020 PMC3408148

[54] Sun D Ding A. MyD88-mediated stabilization of interferon-gamma-induced cytokine and chemokine mRNA. *Nat Immunol.* (2006) 7:375–81. 16491077 10.1038/ni1308

[55] Lai WS Carballo E Thorn JM Kennington EA Blackshear PJ. Interactions of CCCH zinc finger proteins with mRNA. Binding of tristetraprolin-related zinc finger proteins to Au-rich elements and destabilization of mRNA. *J Biol Chem.* (2000) 275:17827–37. 10751406 10.1074/jbc.M001696200

[56] Lu W Ning H Gu L Peng H Wang Q Hou R MCPIP1 selectively destabilizes transcripts associated with an antiapoptotic gene expression program in breast cancer cells that can elicit complete tumor regression. *Cancer Res.* (2016) 76:1429–40. 26833120 10.1158/0008-5472.CAN-15-1115 PMC4794406

[57] Wang Q Ning H Peng H Wei L Hou R Hoft DF Tristetraprolin inhibits macrophage IL-27-induced activation of antitumour cytotoxic T cell responses. *Nat Commun.* (2017) 8:867. 10.1038/s41467-017-00892-y PMC5636828 29021521

