# Supplementary material for: Tristetraprolin Regulates TH17 Cell Function and Ameliorates DSS-Induced Colitis in Mice
Source: Front Immunol. 2020 Aug 14;11:1952. doi: 10.3389/fimmu.2020.01952 (PMC7457025; doi:10.3389/fimmu.2020.01952)
Supplement: Supplementary file 1 [file Presentation_1.pdf]

Supplemental Fig. 1

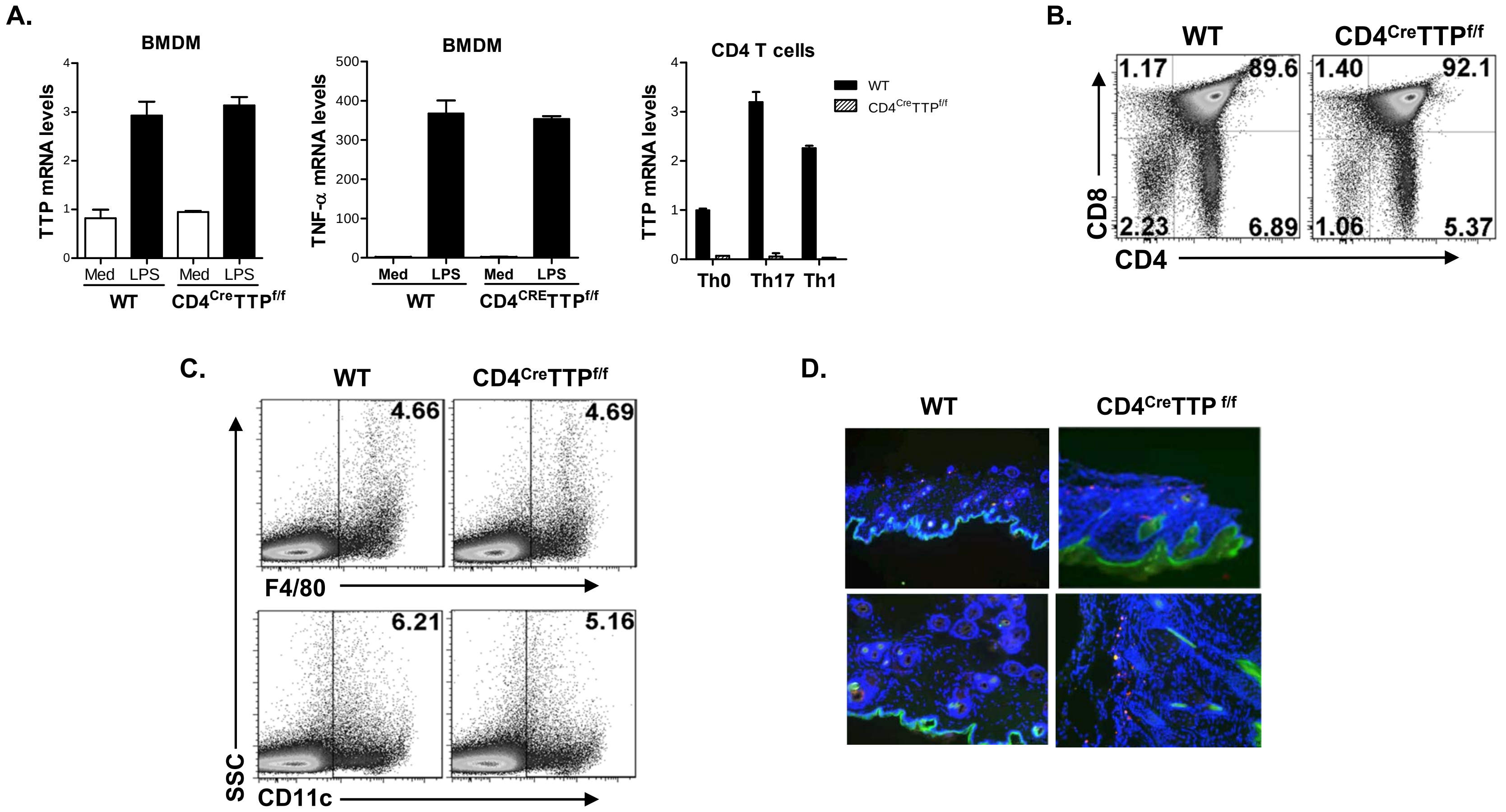

Supplemental Fig. 1. Mice with specific deletion of TTP in T cells have comparable macrophages and DCs. (A) Bone-marrow-derived macrophages (BMDM) were differentiated from bone marrow cells of WT and CD4CreTTPf/f mice with M-CSF (10 ng/ml) for 7 days and then stimulated with LPS (100 ng/ml) for 4 hours, followed by extracting RNA to measure TTP and TNF-α mRNA by qRT-PCR. Naïve CD4 T cells isolated from spleens of WT and CD4CreTTPf/f mice were stimulated with anti-CD3/CD28 Abs or differentiated toward Th17 and Th1 as described in the text and then total RNA was extracted to measure TTP mRNA by qRT-PCR. qRT-PCR data were normalized relative to GAPDH mRNA expression levels in each sample and further normalized to the results from the untreated BMDM (Med, medium alone) or WT Th0 cells, which were set as 1. Data shown are mean plus SD from three independent experiments. Data was compared to which set as 1. (B) Wild type or CD4CreTTPf/f thymocytes were stained with anti-CD3, CD4 and CD8 Abs. FACS was performed by gating on CD3+ T cells. Image shown represent one of two experiments with similar results. (C) Spleen cells from CD4CreTTPf/f mice and WT littermates were stained with anti CD11c and F4/80 Abs. Image shown represent one of two experiments with similar results. (D) Immunofluorescence staining of skin lesions with anti-IL-17A (green) and anti-CD4 (red) antibodies as well as with DAPI (blue).

Supplemental Fig. 2

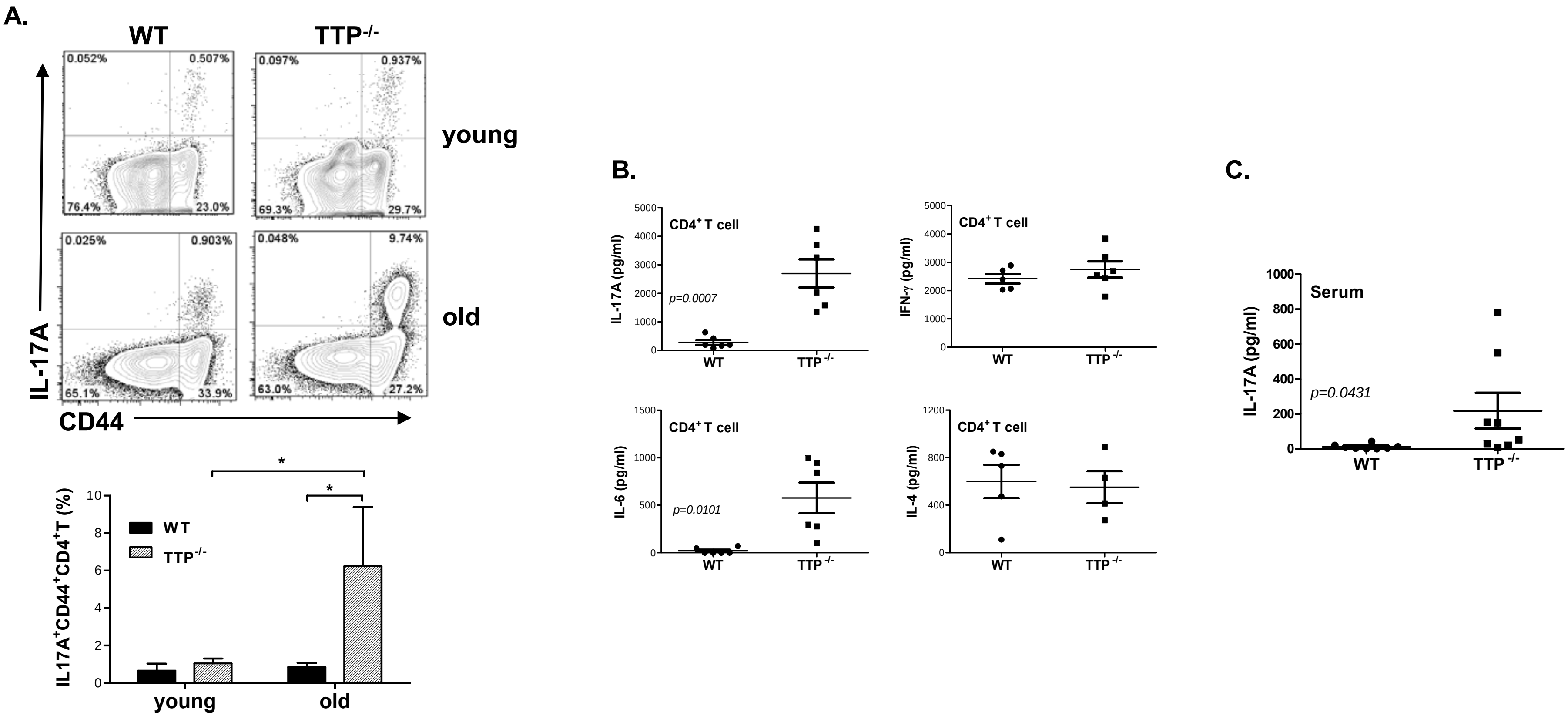

Supplemental Fig. 2. Conventional TTP-/- mice show increased IL-17 and IL-17-producing CD4+ T cells. (A) Splenic CD4+ T cells from TTP-/- mice and WT littermates (young as 3-4 months old and old as 8-10 months old, respectively) were cultured in 96-well plate coated with anti-CD3 (1 µg/ml) and anti-CD28 (1 µg/ml) for 3 days, followed by stimulation with PMA and ionomycin for 4 hours. IL-17A+ and CD44+ cells were detected by flow cytometry gated on CD4+ T cells. Summary data shown are mean plus SD from three experiments. (B) Splenic CD4+ T cells from TTP-/- mice and WT littermates were cultured in 96-well plate coated with anti-CD3 (1 µg/ml) and anti-CD28 (1 µg/ml) for 3 days. IL-17A, IL-6, IL-4 and IFN-γ in supernatants were measured by ELISA. Each dot represents one animal. (C) IL-17A in serum of TTP-/- mice and WT littermates at age of 5-10 months old was detected by ELISA. Each dot represents one animal.

Supplemental Fig. 3

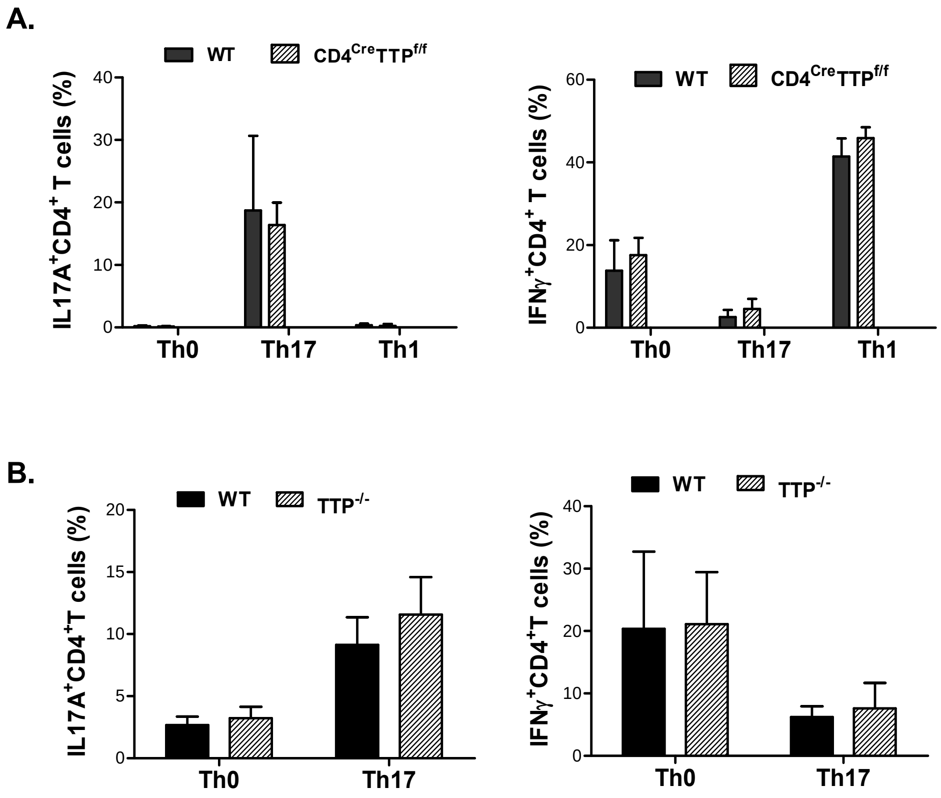

Supplemental Fig. 3. Differentiation of Th17 and Th1 cells are comparable between WT and TTP knockout mice. (A) Naïve CD4+ T cells from CD4CreTTPf/f and WT mice aged 6-8 months were cultured under Th1 or Th17 polarizing conditions in 96-well plate coated with anti-CD3 (2 µg/ml) and anti-CD28 (2 µg/ml) Abs for 3 days before stimulation with PMA and ionomycin for 4 hours. Intracellular IL-17A and IFN-γ in CD4+ T cells were analyzed by FACS. Percentages of Th0, Th17 and Th1 cells were summarized from 3-4 independent experiments. (B) Total CD4+T cells from WT and TTP-/- mice were stimulated with anti-CD3/CD28 Abs under Th17 polarizing conditions for 3 days, rested for 3 days, and then treated with P/I for 4 h, followed by FACS analysis. Percentages of Th0 and Th17 were summarized from independent experiments.

Supplemental Fig. 4

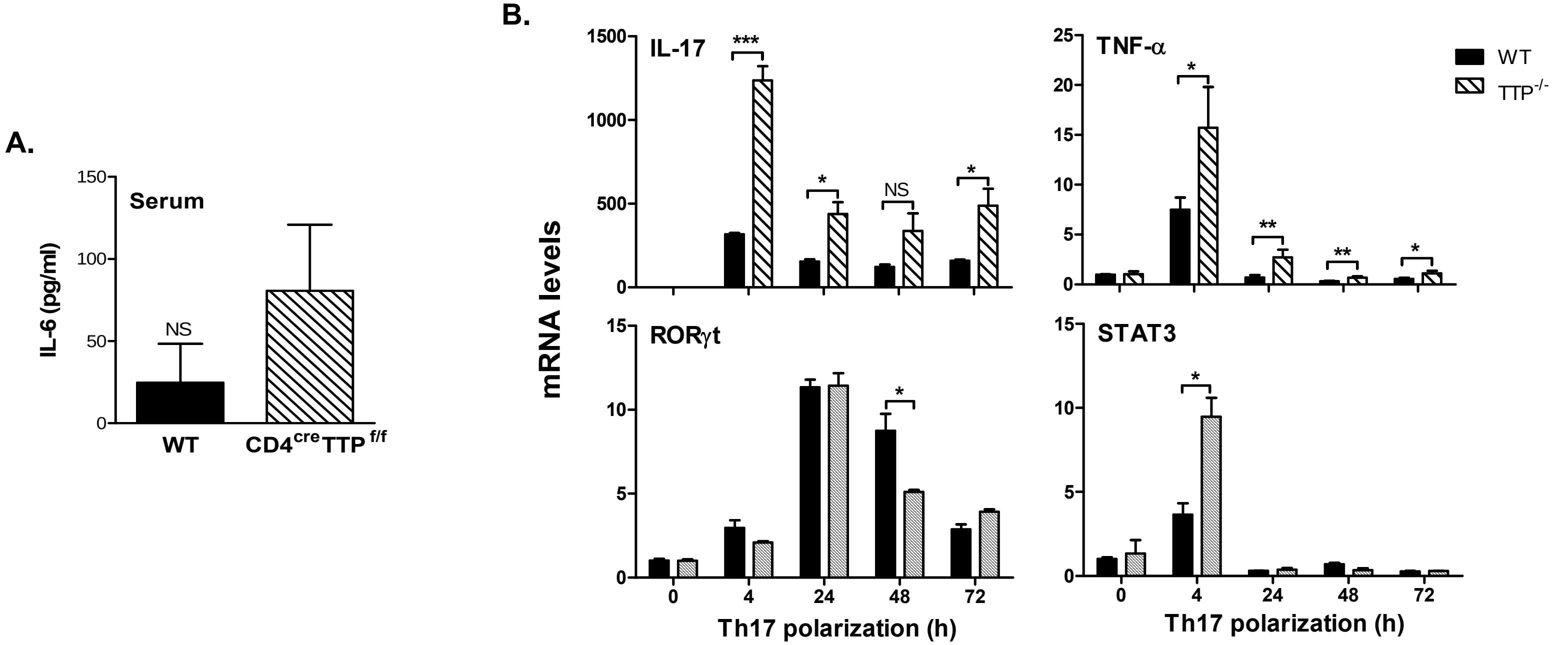

Supplemental Fig. 4. CD4+ T cells deficient of TTP produce more IL-17A. (A) IL-6 in serum of CD4CreTTPf/f mice and WT littermates aged 6-8 months old (three mice in each group) was measured by ELISA. (B) CD4+ T cells of TTP-/- mice and WT littermates aged 3-4 months old were polarized under Th17 conditions for 4, 24, 48, and 72 hours, followed by RNA extraction and measurement of IL-17A, TNF-α, RORγt and STAT3 mRNA expression by real-time q-PCR. PCR data was compared to WT Th17 cells under polarization at 0 hour which set as 1.

Supplemental Fig. 5

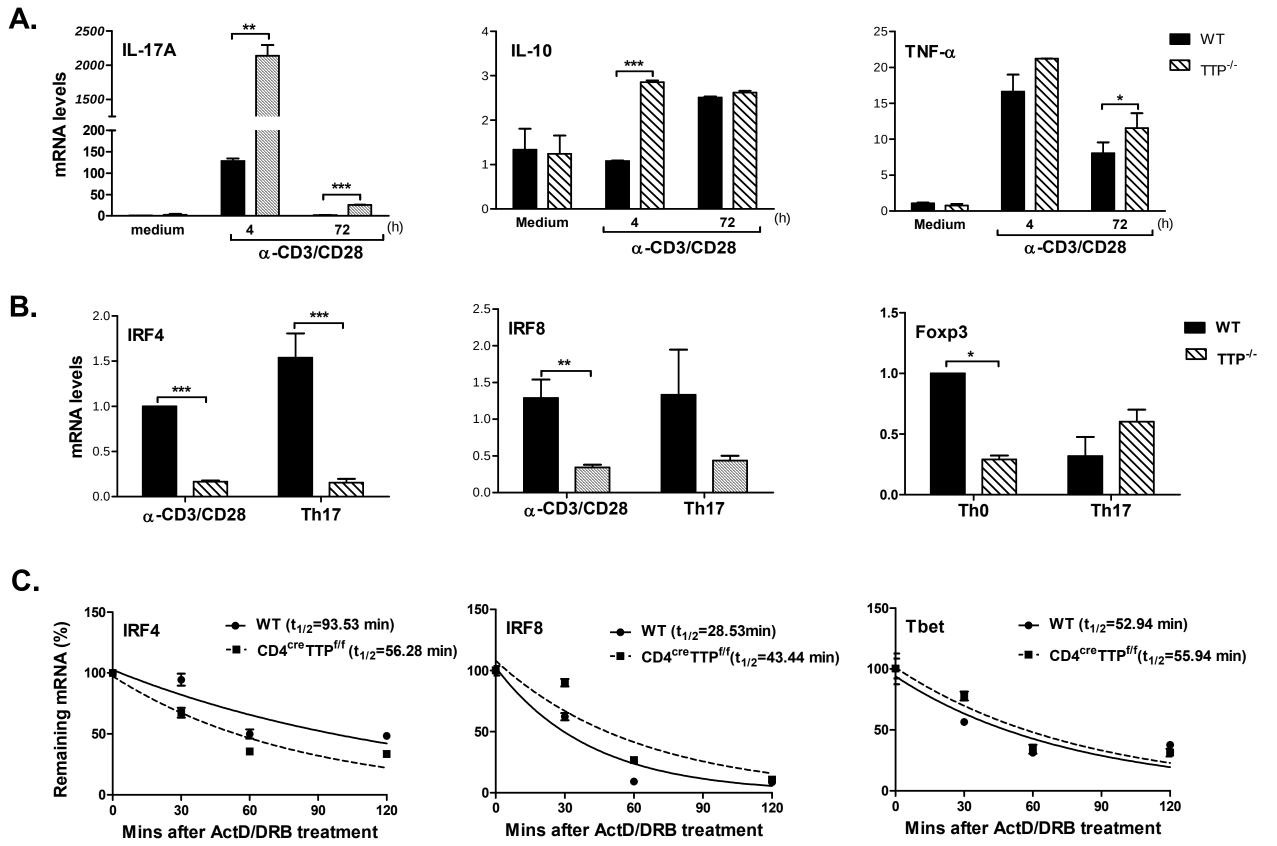

Supplemental Fig. 5. The expression of Th17-related cytokines and transcript factors in CD4 T cells deficient of TTP. (A) CD4+ T cells from TTP-/- mice and WT littermates aged 3-4 months old were stimulated with anti-CD3 (1 µg/ml) and anti-CD28 (1 µg/ml) Abs for 4 and 72 h, respectively, followed by RNA extraction and measurement of IL-17A, TNF-α, and IL-10 mRNA expression by real-time PCR. PCR data were normalized against GAPDH first and then against the levels of IL-17A in WT cells without anti-CD3/CD28 treatment (medium alone). the CT value of (B) The expression of IRF4, IRF8 and Foxp3 in the above CD4 T cells stimulated for 72 hours and in the CD4 T cells under Th17 polarizing condition for 72 hours were detected by real-time PCR. PCR data were normalized against GAPDH first and then against the levels of IL-17A in WT cells stimulated with anti-CD3/CD28 (n=3). (C) Splenic CD4 T cells of CD4CreTTPf/f mice and WT littermates were stimulated under Th17 polarizing conditions for 72 hours, followed by adding Actinomycin D (5 µg/ml) and DRB (10 µg/ml) to block de novo RNA synthesis and then measuring the residue mRNAs of IRF4, IRF8 and Tbet by real-time PCR at the indicated time points.

Supplemental Fig. 6

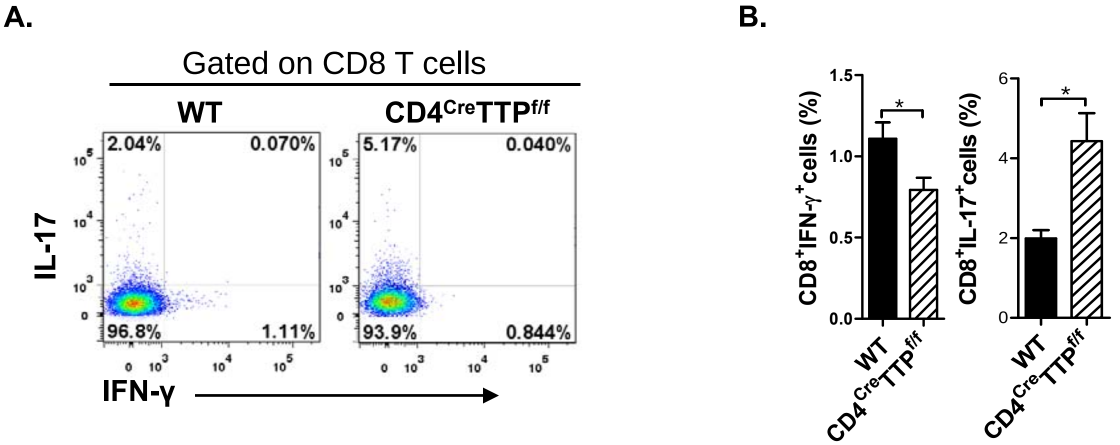

Supplemental Fig. 6. CD8 T cells deficient in TTP secrets more IL-17A. CD8 T cells isolated from spleens of CD4CreTTPf/f mice and WT littermates aged 6-8 months old were stimulated with PMA and ionomycin for 4 hours. Intracellular IL-17A and IFN-γ in CD8+ T cells were analyzed by FACS (A). Percentages of IFN-γ-producing CD8 and IL-17A-producing CD8 T cells from three independent experiments were summarized and compared by t-test (B).
